# Harvesting Mycelial Biomass of Selected Basidiomycetes for Chitosan Biopolymer Extraction

**DOI:** 10.3390/polym15173548

**Published:** 2023-08-26

**Authors:** Ilze Irbe, Laura Andze, Mara Blumfelde, Inese Filipova, Anrijs Verovkins, Juris Zoldners

**Affiliations:** 1 Latvian State Institute of Wood Chemistry, Dzerbenes Iela 27, LV 1006 Riga, Latvia; laura.andze@kki.lv (L.A.); blumfelde007@gmail.com (M.B.); inese.filipova@kki.lv (I.F.); anrijs.verovkins@kki.lv (A.V.); jzoldn@edi.lv (J.Z.); 2 Faculty of Biology, University of Latvia, Raina Bulvaris 19, LV 1586 Riga, Latvia

**Keywords:** basidiomycetes, biopolymers, chitin, chitosan, fungal biomass, solid state fermentation, submerged fermentation

## Abstract

This study investigates the mycelial biomass production and chitosan extraction potential of various Basidiomycota strains, including *Heterobasidion annosum*, *Phanerochaete chrysosporium*, *Pleurotus ostreatus*, *Trametes versicolor*, and *Lentinus lepideus.* Both submerged fermentation (SF) and solid-state fermentation (SSF) methods were employed. The chitosan yield in basidiocarps of *Pleurotus ostreatus*, *Agaricus bisporus*, and *Ganoderma applanatum* was also evaluated as a reference material. The chitosan extracted from fungal cells was characterized using elemental analyses and FTIR spectroscopy. Among the cultivated strains, *P. chrysosporium* exhibited the highest mycelial biomass concentration in SF (1.03 g 100 mL^–1^) after 14 days, while *T. versicolor* achieved the highest biomass concentration in SSF (3.65 g 100 mL^–1^). The highest chitosan yield was obtained from the mycelium of *P. chrysosporium* (0.38%) and *T. versicolor* (0.37%) in shaken SF. Additionally, commercially cultivated *A. bisporus* demonstrated the highest chitosan yield in fungal fruiting bodies (1.7%). The extracted chitosan holds potential as a functional biopolymer additive for eco-friendly materials, serving as an alternative to synthetic wet and dry strength agents in packaging materials.

## 1. Introduction

The fungal cell wall is a complex and well-ordered structure composed of a backbone of polysaccharides, including chitin/chitosan and α/β-glucans, (galacto)-mannans, and glycosylated proteins. Most fungi have a common alkali-insoluble inner wall layer of branched β-(1,3) glucan, β-(1,6) glucan, and chitin but differ substantially in the components in outer layers that are more heterogeneous and tailored to their physiology. The branched β-(1,3):β-(1,6) glucan is bound to proteins and/or other polysaccharides, whose composition may vary with the fungal species [1].

Chitin is one of the most common polysaccharides in nature, found in the majority of fungi, as well as many insects and invertebrates [2]. Chitin makes up about 1–15% of the fungal cell mass with a higher concentration in the cell walls [3,4]. The cell walls and septa of many fungi belonging to the Basidiomycota, Ascomycota, Zygomycota, and Deuteromycota contain chitin to support the strength, shape, and integrity of cell structure. The amount of chitin in the fungal cell wall is specific to species, environmental conditions, and age [5].

Chitosan is less frequent in nature occurring in some fungi (*Mucoraceae*). Chitosan is produced by deacetylation of chitin; in this process, some N-acetylglucosamine moieties are converted into glucosamine units [6]. Commercial chitosan is mainly produced from the chemical deacetylation of chitin from crustacean sources. More recently, chitosan from fungi is gaining interest in the market driven by vegan demands [7].

Chitin and chitosan have many desirable properties such as non-toxicity, biodegradability, bioactivity, biocompatibility, and good adsorption properties which make them suitable alternatives to synthetic polymers [3]. These biopolymers have a large number of potential agricultural, environmental, and biomedical applications [4,7,8]. The global chitosan market size was valued at USD 6.8 billion in 2019 and it is expected to expand at a revenue-based CAGR of 24.7% between 2020 and 2027. The market growth is influenced by the increasing application of the polymer in water treatment and high-value industries such as pharmaceutical, biomedical, cosmetics, and food [7].

Major advantages of chitin and chitosan production from fungal mycelia over crustacean sources include a simpler extraction process, low levels of inorganic materials, needless demineralization, availability with no seasonal or geographic limitations, consistent physico-chemical properties, and low cost of waste management [3]. In this regard, the present work was intended to extract the biopolymers from selected species of Basidiomycota with different environmental distribution, ecological importance, and industrial applications. More specifically, *Heterobasidion annosum* is the most economically important forest pathogen in the Northern Hemisphere causing the root rot in softwoods [9]. *Phanerochaete chrysosporium* is the model white rot (WR) fungus for lignin degradation and is studied for potential applications in bioethanol production [10], the food industry [11], pulp and paper industry [12], bioremediation, and biosorption [13,14]. *Pleurotus ostreatus* is a very important, widely cultivated edible mushroom [15], also studied for its enzymatic application in the pulp and paper industry [16]. *Trametes versicolor* is used in traditional medicine and has shown effects in cancer therapy, antiviral, anti-inflammatory, antimalarial, diabetic, and hepatitis treatment [15,17]. *Lentinus lepideus* is an edible mushroom, cultivated for nutritional and medicinal purposes, including anti-tumor and immunomodulatory activities [18] and bioethanol production [19]. *Agaricus bisporus* is one of the most cultivated edible mushrooms in the world [15], with remarkable nutritional, medicinal, and cosmetic values [20]. The WR fungus *Ganoderma applanatum* has phytochemical properties with potential application in nanotechnological engineering for clinical use [21].

The aim of this study was to obtain the mycelial biomass from fungi *H. annosum*, *P. chrysosporium*, *P. ostreatus*, *T. versicolor*, and *L. lepideus* in two types of cultivation systems (solid and submerged), followed by chitosan extraction, and the biopolymer analyses with analytical methods. The fruiting bodies of *P. ostreatus*, *A. bisporus*, and *G. applanatum* were used as reference material for chitosan content in basidiocarps.

## 2. Materials and Methods

### 2.1. Fungal Material

For determination of mycelial biomass concentration and chitosan yield, the following strains of basidiomycetes were selected: *H. annosum* S.S. V str 28 was provided by the Latvian State Forest Research Institute “Silava”, *P. chrysosporium* LMKK 407 and *P. ostreatus* LMKK 685 were purchased from the Microbial Strain Collection of Latvia (MSCL), *T. versicolor* CTB 863 A and *L. lepideus* BAM 114 were purchased from the Federal Institute for Materials Research and Testing (BAM, Berlin, Germany). To compare the chitosan amount in mycelial biomass and fungal fruiting bodies, the basidiocarps of commercial mushrooms *P. ostreatus* and *A. bisporus* were purchased from a local grocery, and wood destroying fungus *G. applanatum* was collected from a forest ecosystem of Latvia (57 00 N, 25 00 E), Eastern Europe.

### 2.2. Inoculum Preparation

The fungal strains were maintained on malt extract agar (MEA) slants at 6 °C. For inoculum preparation, the mycelium plugs were aseptically transferred to the Petri dishes with MEA medium containing 5% malt extract concentrate, 3% agar (pH 6.0), and cultivated for 14 days in cultivation chamber at temperature of 21 ± 2 °C and relative humidity (RH) 70 ± 5%.

### 2.3. Biomass Cultivation

Two types of fermentation systems were applied: solid-state fermentation (SSF) and submerged fermentation (SF), both of which can be used for biomass production and chitosan isolation.

Initially, the study of mycelium growth dynamics was conducted in SF. A number of 250 mL Erlenmeyer flasks were filled with 100 mL nutrient medium containing (g/L distilled water): glucose—15.0; peptone—3.0; yeast extract—3.0; NaH_2_PO_4_—0.8; K_2_HPO_4_—0.4; MgSO_4_—0.5, pH 6.0. All chemicals for nutrient solution were purchased from Sigma-Aldrich (Steinheim, Germany). Sterilized flasks were inoculated with the 1 cm^2^ mycelium discs and cultivated for 7, 14, 21, 28, and 35 days in rotary shaker incubator (Infors, Bottmingen, Switzerland) at 28 ± 1 °C/150 rpm.

After the growth dynamics experiments, the SSF and SF were applied to produce fungal biomass within a period of 14 days for isolation of chitosan. For SSF, the Petri dishes containing sterile MEA medium (as previously mentioned), were inoculated with the 1 cm^2^ mycelium discs and cultivated in controlled conditions at 21 ± 2 °C/70 ± 5 RH. For stationary culture and shaken SF, the medium and inoculation procedure was identical as described previously. The biomass was cultivated both in stationary culture and shaken culture. After cultivation, the dry biomass was quantified by filtering through the synthetic fabric filter, washing twice with 100 mL deionized water, and drying in an oven at 70 °C until constant weight. Biomass concentration from filtration was expressed in grams of dry weight per 100 mL medium.

### 2.4. Chitosan Extraction

**Figure 1:**
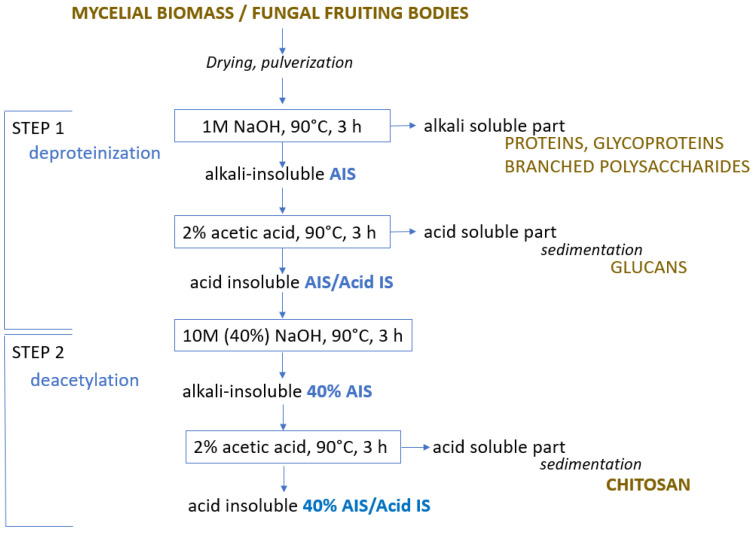
The scheme of two-step chitosan extraction from mycelial biomass or fungal fruiting bodies.

Chitosan from (a) mycelial biomass, obtained after 14 days of cultivation in SSF and shaken SF, as well as from (b) fungal fruiting bodies were extracted via chemical method in a two-step procedure (Figure 1) based on modified method proposed by Mesa Ospina et al. [22]. In Step 1, dried fungal biomass was pulverized and subjected to alkaline treatment with 1M NaOH in the ratio 1:20 (*m*/*v*) at 90 °C for 3 h to remove proteins, glycoproteins, and branched polysaccharides.

Following the treatment, the sample underwent rinsing with deionized water until it achieved a pH of 7.0 and was subsequently filtered. Obtained alkali-insoluble (AIS) material was washed with ethanol and acetone and dried at 100 °C. Then, the sample was heated at 90 °C for 3 h in 2% acetic acid (1:40 (*m*/*v*)). The acid insoluble (AIS/Acid IS) part or chitin-containing part was washed with water, filtered, washed with ethanol and acetone, and dried. The acid-soluble part or glucans were sedimented from the filtrate by changing the acidic pH to pH 9 with NaOH. Sediments were collected on a glass filter, washed with ethanol and acetone, and dried at 100 °C.

In Step 2, deacetylation was implemented. The dried Acid IS part or chitin-containing part was subjected to alkaline treatment with 10M (40%) NaOH in the ratio 1:20 (*m*/*v*) at 90 °C for 3 h. After treatment, the sample was washed with deionized water until a neutral reaction and filtered. Obtained alkali-insoluble (40% AIS) material was washed with ethanol and acetone, and dried at 100 °C. Then the sample was heated at 90 °C for 3 h in 2% acetic acid (1:40 (*m*/*v*)). The acid-insoluble (40% AIS/Acid IS) part was washed with water, filtered, and dried. The acid-soluble part of chitosan was sediment from the filtrate by changing the acidic pH to 9 with NaOH. Sediments were collected on the glass filter, washed with ethanol and acetone, and dried at 100 °C. The yield of each stage was determined gravimetrically. The moisture content of air-dry fungal material was determined according to ISO 18134-32015 [23].

### 2.5. Nitrogen Content

Nitrogen elemental analysis was determined according to the CEN/TS 15104:2011 [24]. Homogenized samples (30 mg) were packed in tin foil, weighed, placed into the carousel of an automatic sample feeder, and analyzed with an Elementar Analysensysteme GmbH (Langenselbold, Germany) Vario MACRO CHNS with a combustion tube temperature of 1150 °C. The original matrix of the sample was destroyed under these conditions through subsequent catalytic reactions.

### 2.6. Fourier Transform Infrared Spectrometry (FTIR)

The milled sample (2 mg) was mixed with 198 mg KBr powder (IR 145 grade, Sigma-Aldrich, St. Louis, MI, USA) and pressed into a small tablet. FTIR spectra were recorded by Nicolet iS50 spectrometer (Thermo Fisher Scientific, Waltham, MA, USA) in the range of 4000–450 cm^−1^ (resolution: 4 cm^−1^, 32 scans per sample).

### 2.7. Statistical Analysis

The statistical analysis was conducted using Excel data analysis. Anova: single factor and correlation were used to explore the relationships between different variables. All statistical tests were conducted at a significance level of α = 0.05, meaning that a *p*-value of 0.05 or less was considered statistically significant and would lead to rejection of the null hypothesis.

## 3. Results and Discussion

### 3.1. Growth Dynamics

The screening of fungal strains at equal cultivation conditions (temperature; rpm) in SF was performed to observe the differences in biomass production with the aim of selecting the optimal time for the collection of biomasses for chitosan extraction.

**Figure 2:**
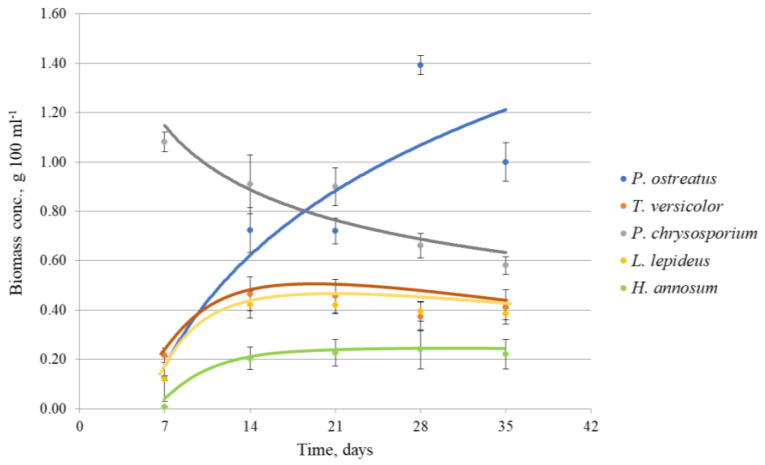
The growth dynamics of fungal strains in liquid shaken culture (SF) in 7–35 days cultivation.

The maximum biomass concentration in liquid SF within 7–35 days varied depending on the fungal strain and duration of cultivation (Figure 2). The highest biomass concentration (1.39 g 100 mL^–1^) was achieved by *P. ostreatus* after long cultivation of 28 days. The highest biomass concentration (1.09 g 100 mL^–1^) in the shortest cultivation time of 7 days demonstrated *P. chrysosporium*. Fungi *L. lepideus* and *T. versicolor* produced lower biomass quantity in the range of 0.4–0.5 g 100 mL^–1^.

*H. annosum* exhibited a remarkably low adaptation to the physical and chemical growth conditions, as evidenced by the lowest biomass concentration achieved under the given cultivation conditions, which measured 0.2 g 100 mL^–1^. *H. annosum* did not show any visible growth even after 7 days of cultivation, and only limited growth was observed by the 14th day. During the subsequent cultivation periods, the growth of the fungus reached a plateau, and the biomass remained relatively constant at a steady level. A similar tendency with a maximum specific growth rate until 14 days was observed in *L. lepideus* and *T. versicolor* cultures. Whereas *P. chrysosporium* reached the maximum growth rate within the first 7 days, with the following gradual decline of biomass production. However, after 14 days, the fungus produced the highest biomass amount among the other strains at the given period. The highest biomass concentration by *P. ostreatus* was achieved after long incubation and cannot be considered economically effective for further experiments. Similar results were obtained by screening more than 50 Basidiomycetes where the majority of species produced higher biomass amounts after 14 days rather than 7 days of incubation [25]. The biomass production of *P. chrysosporium* and *P. ostreatus* showed a statistically significant increase compared to *L. lepideus*, *H. annosum*, and *T. versicolor*. There were no significant differences in biomass production between *P. chrysosporium* and *P. ostreatus*, as well as among *L. lepideus*, *H. annosum*, and *T. versicolor.*

Based on observations of the growth dynamics, a 14-day period was selected as the optimal duration for further solid-state and liquid cultivations, considering the appropriate balance between the amount of fungus and the duration of cultivation.

### 3.2. Biomass Concentration

Observations revealed that in solid culture, the mycelium of all tested strains exhibited radial expansion and formed characteristic aerial mycelium above the substrate. In static liquid culture, the fungi grew as floating pellicles, while in agitated liquid culture, they formed spherical pellets composed of aggregated mycelia. Distinct forms of mycelial growth encounter varying micro-environmental conditions, leading to consequential impacts on fungal physiology and, consequently, fermentation processes [26]. The hyphal branching allows filamentous fungi to fill space in an efficient and appropriate way according to local environmental circumstances. Dense mycelium is produced in nutrient-rich substrate for resource exploitation, whereas hyphae colonize nutrient-poor substrate branches less frequently, producing effuse mycelia [26].

Stationary cultivation in the liquid medium gave the lowest biomass amount in comparison with solid and liquid shaken cultures (Table 1). The exception was *H. annosum* producing the lowest biomass amount in shaken SF. The role of agitation in biomass production was clearly observed in shaken SF. The latter was the most favorable for *P. chrysosporium* with the highest biomass concentration of 1.03 g 100 mL^–1^ among other strains. *P. ostreatus* and *T. versicolor* exhibited the highest biomass concentrations in SSF, measuring 3.04 g 100 mL^–1^ and 3.65 g 100 mL^–1^, respectively. The biomass of these strains exceeded the amount in the shaken culture about five to seven times. The pH after fermentation lay between 3.7–5.7 depending on the fungal strain, and the cultivation method used, with lower values in SSF. For mycelial growth, the initial pH typically ranges from 3.0 to 9.0. However, the specific optimal pH value is dependent on factors such as the fungal species, different strains, and other cultivation conditions including the culture medium [27].

The process of microbial pellet formation and mycelial cell aggregation is influenced by many factors, including the strain used, growth rate, medium composition, shear force, aeration, agitation, and many others. These factors can be divided into three main categories: strain-dependent factors, nutrition-dependent factors, and cultivation conditions [28].

In the present experiments, the medium and cultivation conditions were equal for all tested fungi. The differences in biomass concentration were determined by the strain-specific factors and duration of cultivation (Figure 2). The combination of SSF/nutrition MEA/temperature 21 °C was more favorable for most tested strains than the combination of SF/synthetic medium/temperature 28 °C. An earlier study showed that optimum culture conditions for the *P. chrysosporium* strain were pH 7, temperature 37 °C, and incubation time of 15 days to obtain the maximum amount of the biomass in a stationary liquid medium [29]. Experiments with *P. ostreatus* indicated that the optimal temperature for mycelium growth was 28 °C [30]. The temperature factor can be related to the process of assimilation and translocation of sugar and nitrogen, respiration, and biosynthesis. Most of the fungi are characterized by a preference for a definite temperature regime with subsequent growth enhancement, however, some of them may have the same level of mycelial growth at different temperatures [27].

The effect of cultivation conditions such as temperature, oxygen supply, etc., as well as medium components such as the C source, N source, C/N ratio, and complex organic materials, are reflected in the specific growth rate of the microorganisms [28]. Many microorganisms have strict requirements for certain mineral elements, among them K, P, Mg, Ca, Mo, Fe, Cu, and Zn [31]. In this study, among the applied cultivation methods and fungal strains, the SSF provided the highest biomass concentration (up to 3.65 g 100 mL^–1^) in a 14-day period. Also, the study by Crestini et al. [32] showed that SSF was more efficient in fungal biomass production than SF. The authors reported that SSF of the fungus *Lentinus edodes* yielded a greater biomass concentration (up to 50 times) after 12 days of incubation than that of SF. In our opinion, the SSF method was more time- and labor-consuming in the final biomass collecting phase than SF. These conditions pushed toward the use of SF instead of SSF for biomass production and following chitosan isolation.

### 3.3. Chitin-Containing Part and Chitosan Yield

Table 2 shows the results of chitosan isolation steps—deproteinized fraction (AIS), chitin-containing fraction (AIS/Acid IS), acid-soluble part or beta-glucans, alkali-insoluble (40% AIS) part, acid insoluble (40% AIS/Acid IS) part, and chitosan after deacetylation. The content of chitin-containing parts, glucans, and chitosan within the fungal strains varied depending on the cultivation method. The chitin-containing portion of fungal cell walls accounted for 9% to 27%, with the highest concentration observed in *T. versicolor* cells. In *T. versicolor* culture, it was observed that chitin content depended on the cultivation method with 50% higher content in SSF than in SF. Within the other strains, there was not a distinct difference found between both cultivation methods regarding chitin content.

**Figure 3:**
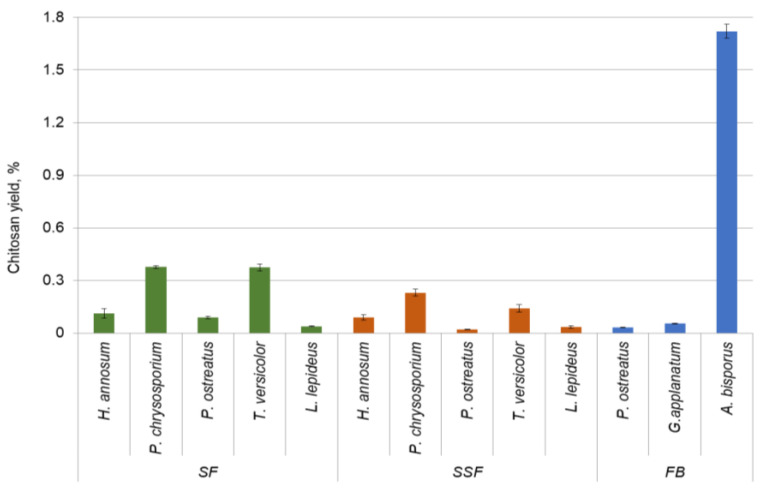
The yield of chitosan from mycelia and fruiting bodies of various fungi. SF = shaken submerged fermentation, SSF = solid state fermentation, FB = fruiting bodies.

When comparing the yield of extracted glucans and chitosan from fungal hyphae, cultivation in SF demonstrated a higher yield of both biopolymers. Glucans at higher amounts of 0.54% and 0.82% were extracted from *P. chrysosporium* and *P. ostreatus* mycelium, respectively. The yield of chitosan from the mycelia and fruiting bodies of various fungi is visually represented in Figure 3. The highest yield of chitosan was obtained from the mycelia of *P. chrysosporium* (0.38%) and *T. versicolor* (0.37%). The cell walls of filamentous fungi are mainly composed of different polysaccharides according to the taxonomic group. They may contain chitin, glucans, mannoproteins, chitosan, and polyglucuronic acid, together with smaller quantities of proteins and glycoproteins [26]. Basidiomycota contains fibrillar polymers chitin and β (1,3)-β (1,6) glucans while Zygomycota is reported to contain chitin and chitosan polymers in the cell wall [26]. Subsequent investigations have provided evidence that chitosan is also found in species belonging to the Ascomycota and Basidiomycota. In the Basidiomycota group, chitosan has been identified in fungi such as *Lentinus edodes* and *Pleurotus sajo-caju* [33].

Chitosan from the fruiting bodies of *P. ostreatus* and *G. applanatum* was obtained at a considerably lower amount than that from *A. bisporus*. Thus, *A. bisporus* with a 1.7% chitosan yield was the most efficient source of biopolymer among the species under study. When comparing the yield of chitosan between the mycelium and fruiting body at the species level, it was found that the amount of chitosan obtained from *P. ostreatus* mycelium in SF was three times higher than that obtained from the fruiting body. Chitosan from *P. ostreatus* mycelium in SSF and the fruiting body was extracted in similar quantities (Table 2). This observation gave evidence that chitosan yield depended on the fungal development stage (mycelium/fruiting body), even more, between the cultivation methods. The quantity and/or quality of chitin and chitosan in the fungal cell wall may change due to environmental and nutritional conditions and the intrinsic characteristics of producing species [33].

The chitosan production from *P. chrysosporium* and *T. versicolor* exhibited a statistically significant increase compared to *L. lepideus*, *H. annosum*, and *P. ostreatus.* A statistically significant correlation between produced biomass and chitosan yield among the tested fungi was not found as p value was above the significance level. For example, the highest biomass concentration of *P. ostreatus* and *T. versicolor* in SSF (Table 1) resulted in a low chitosan yield in this fermentation system (Table 2). Likewise, no correlation was observed between biomass and exopolysaccharide production among the screened basidiomycetes, and in certain cases, a significant decrease in biopolymer content was observed after 14 days of incubation [25]. Fungi are usually harvested at their late exponential growth phase to obtain the maximum yield for chitin and chitosan [31]. In this regard, the present experiments were conducted to screen the specific Basidiomycetes regarding biomass yield in a 14-day period of incubation and its relation to chitosan production.

### 3.4. Nitrogen Content in Fungal Material

Nitrogen was determined to identify and characterize the isolated biopolymers. In Table 3, the results of nitrogen content in raw material, after deproteinization (AIS), in chitin-containing (AIS/Acid IS), glucan, and chitosan fractions are presented. The raw material contained nitrogen from different biopolymers found in fungal cells such as proteins, glycoproteins, and chitin. Nitrogen is structurally and functionally linked with fungal cellular functions as organic amino nitrogen in proteins and enzymes [26]. The highest total nitrogen was found in the mycelia of *H. annosum*, *T. versicolor*, and *L. lepideus* in liquid cultures reaching more than 6%. These strains in solid cultures contained a two- to three-times lower amount of nitrogen.

After protein removal, the nitrogen remained in the structural components of the cell wall including chitin. The nitrogen amount in the chitin-containing fraction depended on the cultivation method with a higher content in the liquid medium. In fruiting bodies, the highest nitrogen percentage in the chitin-containing fraction was determined for *A. bisporus*. Small amounts (0.14–0.54%) of nitrogen were found in the glucan fraction of the mycelial biomass and fruiting bodies. This can be explained by the small amount of chitin released together with glucans in the form of chitin–glucan complexes [34].

The nitrogen in isolated chitosan comprised over 6% both in the mycelium and fruiting bodies depending on the fungal species. This is close to the actual nitrogen content of chitosan (6.6–8.2%) reported in the literature [35]. Alvarenga [36] found that the percentage of nitrogen in fully deacetylated chitosan was 8.695, and in fully acetylated chitin—6.896. In this study, the nitrogen content in the chitosan fraction was lower than in pure chitosan, because of the small amount of chitin–glucan and chitosan–glucan complexes remaining in the chitosan fraction [37]. In order to completely break down the chitin–glucan complexes, it would be necessary to use enzymatic treatment [38]. The highest nitrogen content was determined in fungal samples containing the highest chitosan yield, i.e., the mycelium of *P. chrysosporium* and *T. versicolor*, and the fruiting body of *A. bisporus* (Table 2).

### 3.5. Characterization of Fungal Chitosan by Fourier Transform Infrared Spectra

FTIR method was used to identify the eluted chitosan samples and compare them with commercial chitosan (Sigma-Aldrich, USA). The characteristic peaks used for the identification of chitosan are as follows: the peak at 1376 cm^−1^ corresponds to amide III, the peak at 1544 cm^−1^ is associated with the amino group NH_2_, the peak at 1647 cm^−1^ indicates amide I, the peak at 2914 cm^−1^ represents the stretching band of C–H and –C=O in the amide group CONH–R of the polymers [35], and the peak at 3400 cm^−1^ indicates the presence of -OH stretching and symmetric vibrations of the amine N-H group [39].

**Figure 4:**
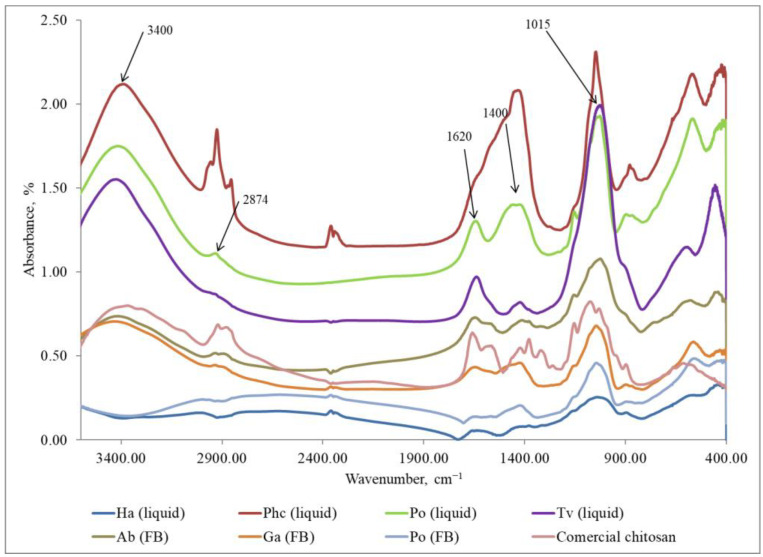
FTIR spectra of the samples extracted in Step 1 (glucans) from the fungal mycelium (liquid, submerged fermentation) and fruiting bodies (FB) compared with the commercial chitosan. Ha (liquid) = *H. annosum*; Phc (liquid) = *P. chrysosporium*; Po (liquid) = *P. ostreatus*; Tv (liquid) = *T. versicolor*; Ab (FB) = *A. bisporus*; Ga (FB) = *G. applanatum*; Po (FB) = *P. ostreatus;* commercial chitosan (Sigma-Aldrich).

The results (Figure 4) provided confirmation that the samples isolated from fungi in Step 1 did not exhibit the characteristic FTIR peaks of chitosan. Instead, the peaks observed were attributed to other polysaccharides. Characteristic FTIR spectra of polysaccharides in the region of 3300–3500 cm^−1^ indicate the stretching vibrations of OH groups. The peak at 2874 cm^−1^ corresponds to the stretching vibrations of the C–H groups and 1662 cm^−1^ is connected with deformational vibrations of OH-groups of bound water. The FTIR peaks in the region of 1400–1500 cm^−1^ show CH_2_ bending. The FTIR bands in the range of 1500–1200 cm^−1^ are sensitive to chemical and molecule structural transformations. The region of 1000–1200 cm^−1^ is connected with stretching C–O–C and C–O vibrations [40,41]. It can be concluded that in Step 1, glucans, together with other compounds, were released from mycelium and fruiting bodies [42,43]. The FTIR method is sensitive to the position and anomeric configuration of glycosidic linkages in glucans. Fruiting bodies of mushrooms contain two main types of glucans: branched (1 → 3) (1 → 6)-β-D-glucan and linear (1 → 3)-α-D-glucan [44].

**Figure 5:**
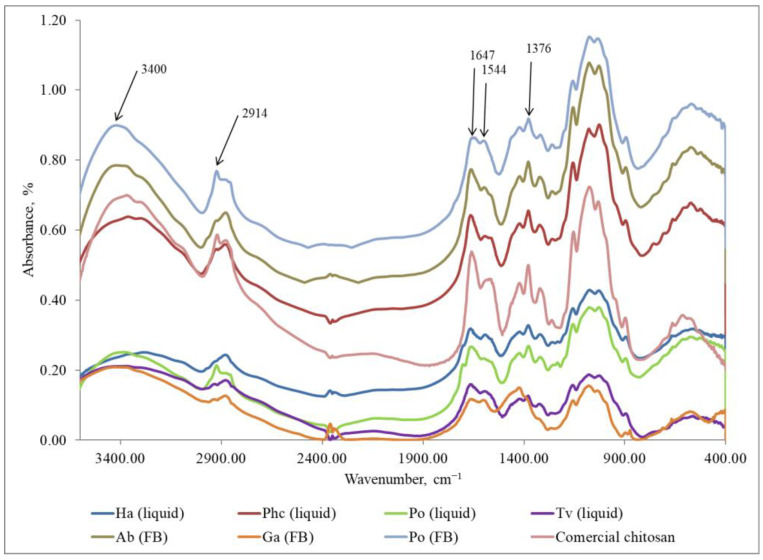
FTIR spectra of the samples extracted in Step 2 (chitosan) from fungal mycelium (liquid, submerged fermentation) and fruiting bodies (FB) compared with the commercial chitosan. Ha (liquid) = *H. annosum*; Phc (liquid) = *P. chrysosporium*; Po (liquid) = *P. ostreatus*; Tv (liquid) = *T. versicolor*; Ab (FB) = *A. bisporus*; Ga (FB) = *G. applanatum*; Po (FB) = *P. ostreatus*; commercial chitosan (Sigma-Aldrich).

The FTIR spectra of extracted chitosan coincided with the spectrum of commercially produced chitosan (Figure 5). All peaks identifying chitosan were visible in the FTIR spectra. A similar result was obtained in research by Mesa Ospina et al. [22] where chitosan was isolated from the *Ganoderma lucidum* basidiomycete through the deacetylation of chitin. The FTIR spectra of chitosan obtained from this mushroom had a significant similitude with commercial chitosan.

Based on the presented results (Table 2), the chitosan isolation method is applicable to obtain a pure biopolymer (Figure 5) from fungal material with the highest amount extracted from cultivated mycelium of *P. chrysosporium* and fruiting body of *A. bisporus.* Despite the presence of hydrophilic amine and -OH groups, films of chitosan exhibit hydrophobic characteristics [45]. The solubilized chitosan films can be considered for food-contact purposes [46,47] as they can resist wetting. Chitosan as a functional biopolymer additive is suggested as a substitute for synthetic wet and dry strength agents in packaging materials.

## 4. Conclusions

The investigation of fungi through SF revealed that the peak biomass concentration during the 7–35-day interval was contingent upon both the fungal strain and the duration of cultivation. Among the cultivation periods tested, a 14-day duration demonstrated the optimal correlation between fungal strain and biomass concentration. Regarding the cultivation techniques employed and the specific fungal strains used, it was observed that SSF yielded a greater biomass quantity compared to SF.

With the selected two-step chemical extraction method, it was possible to isolate chitosan from cultured fungal mycelia as well as from collected fruiting bodies. The content of chitin-containing part/chitosan within fungal species varied depending on the method of cultivation.

No statistically significant correlation was found between produced biomass and chitosan yield among the tested fungi. The highest chitosan yield among the cultivated strains was obtained from the mycelia of *P. chrysosporium* and *T. versicolor* in SF. Thus, these strains have potential applications in biotechnological processes for chitosan production. Among the fungal fruiting bodies, commercially cultivated *A. bisporus* exhibited the highest chitosan yield. Consequently, the remains (stalks) of *A. bisporus*, generated during food production, hold promise as a viable resource for chitosan isolation. The extracted chitosan, as a functional biopolymer additive, could potentially replace synthetic wet and dry strength agents in packaging materials.

## Figures and Tables

**Table 1:** Fungal biomass concentration in solid (SSF), shaken submerged (SF), and stationary submerged (SF) culture after 14 days cultivation (*n* = 3).

Fungus	Dry Biomass (g 100 mL^–1^)		
	SSF	Shaken SF	Stationary SF
*P. ostreatus*	3.04 ± 0.39	0.63 ± 0.05	0.09 ± 0.03
*T. versicolor*	3.65 ± 0.66	0.48 ± 0.04	0.39 ± 0.03
*P. chrysosporium*	0.79 ± 0.21	1.03 ± 0.05	0.19 ± 0.00
*L. lepideus*	1.85 ± 0.29	0.40 ± 0.02	0.02 ± 0.01
*H. annosum*	0.75 ± 0.04	0.20 ± 0.03	0.36 ± 0.14

**Table 2:** Sample yield (%) in different stages of chitosan isolation from mycelial biomass obtained in shaken submerged fermentation (SF), solid-state fermentation (SSF), and fruiting bodies (FB). AIS = alkali-insoluble part; AIS/Acid IS = acid insoluble or chitin-containing fraction.

Fungus	Cultivation	Moisture	AIS	Chitin-Containing Part AIS/Acid IS)	Glucans	40% AIS	40% AIS/Acid IS	Chitosan
*H. annosum*	SF	6.8	11.6 ± 0.2	10.0 ± 0.6	0.12 ± 0.01	6.3 ± 0.2	2.7 ± 0.2	0.11 ± 0.04
	SSF	6.1	12.4 ± 0.3	9.4 ± 0.3	0.23 ± 0.04	5.0 ± 0.3	2.5 ± 0.3	0.09 ± 0.02
*P. chrysosporium*	SF	8.2	18.5 ± 0.7	13.7 ± 0.4	0.54 ± 0.03	4.4 ± 0.7	2.1 ± 0.3	0.38 ± 0.01
	SSF	9.1	15.5 ± 0.5	12.7 ± 0.6	0.26 ± 0.02	3.4 ± 0.4	1.9 ± 0.4	0.23 ± 0.02
*P. ostreatus*	SF	7.3	15.7 ± 0.4	13.3 ± 0.4	0.82 ± 0.03	6.7 ± 0.6	4.2 ± 0.2	0.09 ± 0.01
	SSF	6.8	18.4 ± 0.3	13.8 ± 0.3	0.28 ± 0.01	2.2 ± 0.3	1.3 ± 0.1	0.021 ± 0.002
*T. versicolor*	SF	8.4	15.4 ± 0.6	12.8 ± 0.3	0.17 ± 0.0.7	5.5 ± 0.2	3.3 ± 0.6	0.37 ± 0.02
	SSF	7.3	30.9 ± 0.3	26.8 ± 0.5	0.10 ± 0.05	17.5 ± 0.8	15.2 ± 0.4	0.14 ± 0.02
*L. lepideus*	SF	8.9	11.3 ± 0.8	10.1 ± 0.7	0.07 ± 0.03	5.2 ± 0.2	2.5 ± 0.2	0.039 ± 0.003
	SSF	9.8	9.9 ± 0.2	8.9 ± 0.4	0.06 ± 0.02	4.8 ± 0.4	4.5 ± 0.4	0.035 ± 0.006
*P. ostreatus*	FB	5.0	21.42 ± 0.4	–	0.04 ± 0.01	–	–	0.032 ± 0.002
*G.applanatum*	FB	8.3	48.05 ± 0.5	–	0.05 ± 0.04	–	–	0.054 ± 0.003
*A. bisporus*	FB	6.8	15.6 ± 0.2	14.3 ± 0.2	0.11 ± 0.01	8.4 ± 0.4	2.5 ± 0.2	1.72 ± 0.04

**Table 3:** Nitrogen content in different stages of chitosan isolation from mycelial biomass obtained in (a) shaken submerged cultivation (SF), solid-state fermentation (SSF), and (b) fruiting bodies (FB). AIS = alkali-insoluble part; AIS/Acid IS = acid insoluble or chitin-containing fraction.

Fungus	Nitrogen Content (%)					
	Cultivation	Raw Material	AIS	Chitin-Containing Part (AIS/acid IS)	Glucans	Chitosan
*H. annosum*	SF	6.41 ± 0.07	3.48 ± 0.05	4.15 ± 0.06	0.54 ± 0.01	4.02 ± 0.02
	SSF	3.38 ± 0.07	2.30 ± 0.06	2.99 ± 0.06	0.30	-
*P. chrysosporium*	SF	5.30 ± 0.03	2.58 ± 0.20	2.88 ± 0.25	0.31 ± 0.07	6.00 ± 0.04
	SSF	1.59 ± 0.01	1.52 ± 0.01	1.96 ± 0.08	0.53	5.43
*P. ostreatus*	SF	5.78 ± 0.02	2.93 ± 0.02	3.09 ± 0.02	0.38 ± 0.02	2.77 ± 0.03
	SSF	1.40 ± 0.28	1.09 ± 0.05	1.36 ± 0.01	0.27	-
*T. versicolor*	SF	6.15 ± 0.25	2.36 ± 0.02	3.04 ± 0.20	0.14 ± 0.02	6.16
	SSF	1.62 ± 0.03	1.01 ± 0.01	1.39	-	5.29
*L. lepideus*	SF	7.29 ± 0.07	2.63 ± 0.13	-	0.17 ± 0.02	-
	SSF	1.39 ± 0.01	1.01 ± 0.15	-	-	-
*P. ostreatus*	FB	4.55 ± 0.05	1.35 ± 0.02	1.37 ± 0.02	0.14 ± 0.01	3.80 ± 0.01
*G. applanatum*	FB	3.07 ± 0.01	3.32 ± 0.03	3.01 ± 0.01	-	4.82 ± 0.04
*A. bisporus*	FB	3.95 ± 0.02	4.02 ± 0.02	4.25 ± 0.01	0.43	6.24 ± 0.04

## Data Availability

Data will be available upon reasonable request.
